# Quantitative Assessment of Occipital Metabolic and Energetic Changes in Parkinson’s Patients, Using In Vivo ^31^P MRS-Based Metabolic Imaging at 7T

**DOI:** 10.3390/metabo11030145

**Published:** 2021-03-01

**Authors:** Xiao-Hong Zhu, Byeong-Yeul Lee, Paul Tuite, Lisa Coles, Abhishek G. Sathe, Chi Chen, Jim Cloyd, Walter C. Low, Clifford J. Steer, Wei Chen

**Affiliations:** 1 Center for Magnetic Resonance Research, Department of Radiology, University of Minnesota, Minneapolis, MN 55455, USA; catchjoy73@gmail.com; 2 Department of Neurology, University of Minnesota, Minneapolis, MN 55455, USA; tuite002@umn.edu; 3 Department of Experimental and Clinical Pharmacology, University of Minnesota, Minneapolis, MN 55455, USA; durh0016@umn.edu (L.C.); sathe134@umn.edu (A.G.S.); cloyd001@umn.edu (J.C.); 4 Department of Food Science and Nutrition, University of Minnesota, Minneapolis, MN 55455, USA; chichen@umn.edu; 5 Department of Neurosurgery, University of Minnesota, Minneapolis, MN 55455, USA; lowwalt@umn.edu; 6 Departments of Medicine and Genetics, Cell Biology and Development, University of Minnesota, Minneapolis, MN 55455, USA; steer001@umn.edu

**Keywords:** cerebral ATP energy metabolism, human brain, in vivo ^31^P MRS-based metabolic imaging, neurodegenerative disease, ursodeoxycholic acid (UDCA)

## Abstract

Abnormal energy metabolism associated with mitochondrial dysfunction is thought to be a major contributor to the progression of neurodegenerative diseases such as Parkinson’s disease (PD). Recent advancements in the field of magnetic resonance (MR) based metabolic imaging provide state-of-the-art technologies for non-invasively probing cerebral energy metabolism under various brain conditions. In this proof-of-principle clinical study, we employed quantitative ^31^P MR spectroscopy (MRS) imaging techniques to determine a constellation of metabolic and bioenergetic parameters, including cerebral adenosine triphosphate (ATP) and other phosphorous metabolite concentrations, intracellular pH and nicotinamide adenine dinucleotide (NAD) redox ratio, and ATP production rates in the occipital lobe of cognitive-normal PD patients, and then we compared them with age-sex matched healthy controls. Small but statistically significant differences in intracellular pH, NAD and ATP contents and ATPase enzyme activity between the two groups were detected, suggesting that subtle defects in energy metabolism and mitochondrial function are quantifiable before regional neurological deficits or pathogenesis begin to occur in these patients. Pilot data aiming to evaluate the bioenergetic effect of mitochondrial-protective bile acid, ursodeoxycholic acid (UDCA) were also obtained. These results collectively demonstrated that in vivo ^31^P MRS-based neuroimaging can non-invasively and quantitatively assess key metabolic-energetic metrics in the human brain. This provides an exciting opportunity to better understand neurodegenerative diseases, their progression and response to treatment.

## 1. Introduction

Energy metabolism is a fundamental process of life, and adenosine triphosphate (ATP) produced by mitochondrial oxidative phosphorylation (OXPHOS) is the main source of chemical energy for all cellular activities in the brain [1,2,3]. Due to various cellular defects or mitochondrial abnormalities, brain cells may fail to meet the energy requirements, which can lead to cerebral dysfunction and neurodegeneration [4]. A growing body of evidence suggests that neurodegenerative diseases such as Alzheimer’s disease (AD) and Parkinson’s disease (PD) are associated with abnormal mitochondrial function and impaired cerebral energy metabolism, which evolve over time and play a critical role in the pathogenesis and progression of the disease [5,6,7]. Therefore, monitoring bioenergetic changes in diseased brains could be an effective way to study neurodegeneration, its progression and the effectiveness of treatment modalities. However, direct and quantitative measurement of bioenergetics in human brain is challenging due to the lack of appropriate neuroimaging tools.

Positron emission tomography (PET) has been established to evaluate regional brain glucose or oxygen utilization, neurochemical changes and inflammation in AD and PD brains [8,9,10,11], but it is limited in assessing mitochondrial enzymatic activities and ATP bioenergetics. Magnetic resonance (MR) spectroscopy (MRS) is capable of non-invasively identifying neurochemical information via detection of major metabolites in the living human brain [12,13,14,15,16]; and it has been applied to investigate abnormal cerebral metabolism and bioenergetics in human patients [17,18,19,20,21]. In particular, in vivo ^31^P MRS can directly detect constituents of endogenous phosphorous metabolites, including ATP, phosphocreatine (PCr), inorganic phosphate (Pi), phosphoethanolamine (PE) and glycerophosphocholine (GPC), as well as intracellular pH and free magnesium content ([Mg^2+^]). It should, therefore, provide an ideal tool for assessing the link between energy failure and neurodegenerative diseases [4,22]. Nevertheless, only a small number of such studies have been reported and most of them were performed on relatively low-field (≤3 Tesla (T)) MR imaging (MRI) scanners with limited signal-to-noise ratio (SNR) and sub-optimal spectral quality [18,23,24,25,26,27,28,29,30,31]. In addition, although absolute quantification is desired, it is not easy to accomplish [32]; and thus, most previous studies only provide qualitative information by reporting signals or concentration ratios between different metabolites. This makes it difficult to interpret results, especially in the absence of any reliable internal metabolite reference in the diseased state.

With recent technology advancement and increased availability of ultrahigh-field (UHF, ≥7T) MRI scanners, the advantages of in vivo ^31^P MRS at UHF have been demonstrated and well recognized. The substantial SNR gain and improved spectral resolution, as well as markedly shortened longitudinal relaxation time (T_1_) of phosphorous metabolites at UHF have greatly improved the sensitivity and accuracy of the in vivo ^31^P MRS measurement with significantly reduced measurement time [33,34], which is critical for patient studies. Furthermore, we have developed several novel in vivo ^31^P MRS based neuroimaging techniques that can measure and quantify not only the concentration of major phosphorous metabolites, but also cerebral ATP production rates and redox state of nicotinamide adenine dinucleotide (NAD) in animal and human brains [35,36,37,38,39]. An experimental protocol for quantifying a range of bioenergetic and neurophysiological parameters has also been established for human brain application using a radiofrequency (RF) surface coil [40]. Thus, it is possible to provide essential parameters and quantities of interest in *absolute units*, so that the bioenergetic state of the human brain in different locations, times and/or conditions can be directly evaluated and compared. These capabilities open new opportunities for studying abnormal brain metabolism and bioenergetics in human patients diagnosed with neurodegenerative diseases.

In this work, we conducted a proof-of-principle study on patients with mild to moderate Parkinson’s disease using a 7T human MRI scanner. We selected the occipital lobe of PD patients with normal cognitive function as the target area of this study based on the considerations that (i) PD is a neurodegenerative disorder affecting many brain regions; (ii) the current ^31^P MRS-based neuroimaging technology can achieve the best detection sensitivity in the cortical brain regions; and (iii) we intended to test the feasibility of detecting subtle metabolic and energetic changes at an early stage of neurodegeneration. In addition, using the same methodology, pilot data were obtained from several PD patients to assess the potential bioenergetic effects of the mitochondrial-protective bile acid ursodeoxycholic acid (UDCA) [41,42,43], a Food and Drug Administration (FDA)-approved drug for treating primary biliary cholangitis.

## 2. Results

### 2.1. Characterization of the Study Participants

A total of 19 cognitively normal, mild to moderate PD patients and an equal number of healthy control (CT) subjects participated in this study. They were recruited into two separate cohorts for different ^31^P MRS-based metabolic imaging measurements (see details below). The characteristics of the participants are summarized in Table 1. The age and sex were well matched between the corresponding PD and CT groups, and their MoCA scores were not significantly different. Table 1 also includes the UPDRS and MoCA scores of the patients before and after UDCA treatment. Although a slight improvement with UDCA was detected, the data did not reach a significant level due to the small sample size.

### 2.2. Cerebral Phosphorous Metabolite Profiles of PD Patients and Controls

**Figure 1:**
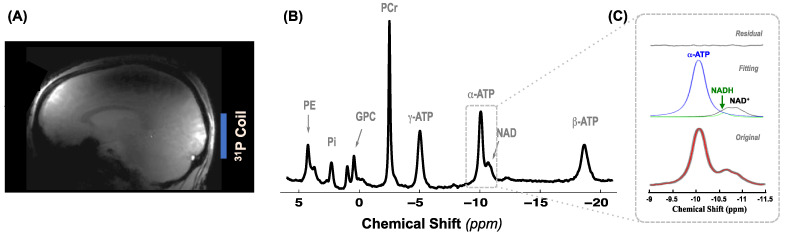
(**A**) ^1^H MR image (*sagittal orientation*) of a subject brain showing the size and location of the ^31^P surface coil used in the study; (**B**) a representative ^31^P MR spectrum obtained from a PD patient; and (**C**) expanded original and fitted spectra covering the chemical shift range of a-ATP, NAD^+^ and NADH (gray trace: original data, red trace: spectral fitting, blue/black/green traces: decomposed a-ATP, NAD^+^ and NADH signals).

To accurately determine the contents of phosphorous metabolites and other key physiological parameters in healthy and diseased brains, we acquired high quality ^31^P spectra from each participant at UHF of 7T. Figure 1A indicates the position of the ^31^P coil relative to human brain in a ^1^H MRI; and Figure 1B shows a typical ^31^P MR spectrum obtained from the occipital lobe of a representative patient. Excellent sensitivity, spectral resolution and fitting quality are evident from Figure 1C, which displays original and spectral fitting of the α-ATP, oxidized (NAD^+^) and reduced (NADH) NAD resonance signals with a very small residual. The superb quality of the ^31^P spectral data is also confirmed by the high SNR and narrow linewidth (LW) of the PCr resonance peak and their consistency as determined in Cohort I PD (SNR_PCr_ = 268 ± 44 and LW_PCr_ = 20 ± 1 Hz, *n* = 8) and CT (SNR_PCr_ = 273 ± 25 and LW_PCr_ = 21 ± 5 Hz, *n* = 8) brains with *p*-values of 0.79 for SNR_PCr_ and 0.38 for LW_PCr_, respectively.

**Figure 2:**
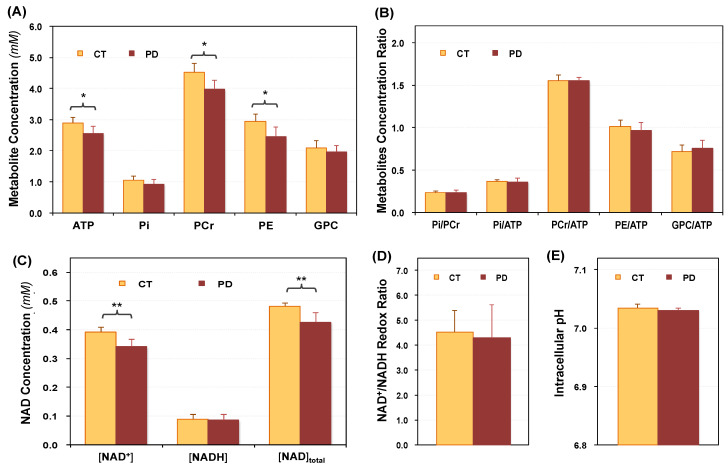
Occipital phosphorous metabolites profile in Parkinson’s patients (PD, *n* = 8) and age/gender-matched healthy controls (CT, *n* = 8) of Cohort I. (**A**) Metabolite concentrations of ATP, Pi, PCr, PE, GPC and (**B**) their ratios; (**C**) intracellular NAD^+^, NADH and total NAD contents, (**D**) NAD^+^/NADH redox ratio; and (**E**) intracellular pH are presented. * *p* < 0.01 and ** *p* < 0.001 indicate that statistic significant differences were detected with 2-tailed student *t*-test; and all data are presented as Mean ± SD.

Figure 2 summarizes the results obtained from Cohort I and indicates that the concentrations of ATP, PCr, PE, NAD^+^ and total NAD (=[NAD^+^] + [NADH]) in the occipital lobe of the PD patients were lower than those of healthy controls, and the differences were statistically significant (*p* < 0.01, *n* = 8; Figure 2A,C). On the other hand, no significant difference in the metabolites ratio of PCr/ATP, Pi/ATP, Pi/PCr, PE/ATP and GPC/ATP between the two groups was detected (*p >* 0.2, *n =* 8; Figure 2B). The NAD^+^/NADH redox ratio (RX, Figure 2D) and intracellular pH (Figure 2E) were slightly lower in the PD brains (pH_PD_ = 7.031 ± 0.003, RX_PD_ = 4.29 ± 1.33, *n* = 8) than those of CT (pH_CT_ = 7.035 ± 0.006, RX_CT_ = 4.52 ± 0.87, *n* = 8), which did not reach a statistical significance (the *p* values were 0.09 and 0.68, respectively). The group-averaged concentrations of NAD^+^ ([NAD^+^]_PD_ = 0.34 ± 0.03 millimolar (mM), [NAD^+^]_CT_ = 0.39 ± 0.02 mM, *n* = 8, *p* = 0.0002), NADH ([NADH]_PD_ = 0.09 ± 0.02 mM, [NADH]_CT_ = 0.09 ± 0.02 mM, *n* = 8, *p* = 0.66), and total NAD ([NAD_total_]_PD_ = 0.43± 0.03 mM, [NAD_total_]_CT_ = 0.48 ± 0.01 mM, *n* = 8, *p* = 0.0006) are reported herein; and the distributions of the individual values of NAD^+^, NADH, total NAD and RX obtained from all participants in Cohort I can be found in Figure S1. The NAD molar concentrations presented here were calculated after correcting the saturation effect caused by a short repetition time used in acquiring the in vivo ^31^P MRS data, which are slightly higher than those reported in earlier studies. However, this correction did not affect the RX values because both NAD^+^ and NADH levels were corrected in the same way [37,39].

**Figure 3:**
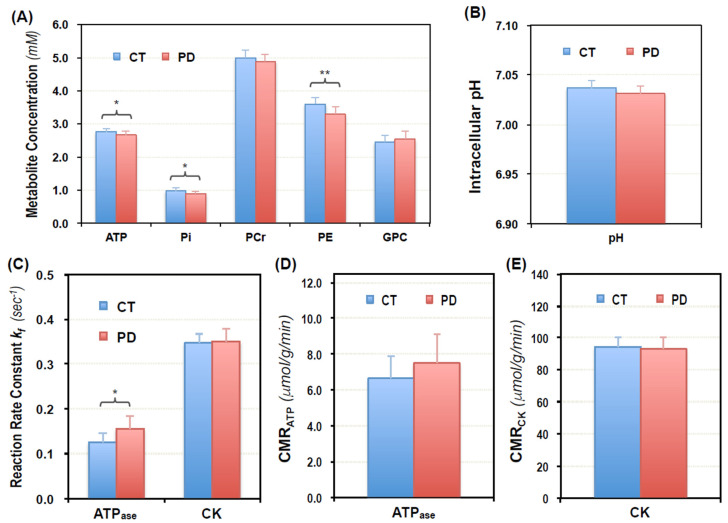
Summary of phosphorous metabolites concentration (**A**), intracellular pH (**B**), forward rate constant (**C**) and cerebral metabolic rate of ATPase (**D**) and CK (**E**) reactions measured in the occipital lobe of PD patients (PD, *n* = 11) and age-/gender-matched control subjects (CT, *n* = 11) of Cohort II. All data are presented as Mean ± SD. * *p* < 0.05 and ** *p* < 0.005 indicate that significant differences were detected with 2-tailed Student *t*-test.

**Figure 4:**
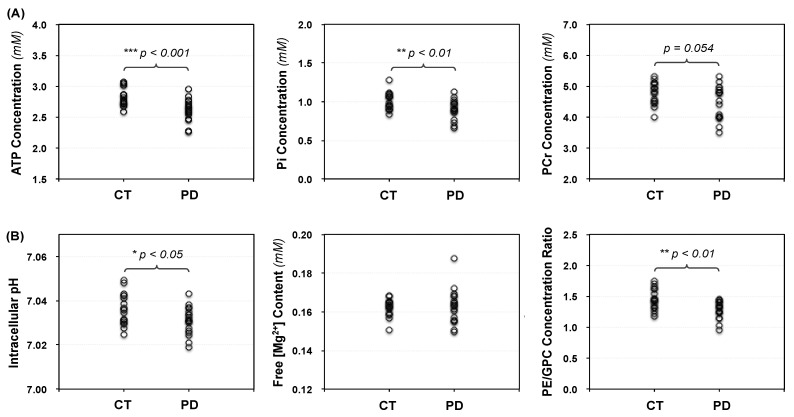
Summarized ATP, PCr and Pi concentrations (**A**), intracellular pH, free [Mg^2+^] and PE/GPC ratio (**B**) obtained from all participants in Cohorts I–II showing the distribution of the these values from individual PD patients (*n* = 19) and healthy controls (*n* = 19). * *p* < 0.05, ** *p* < 0.01 and *** *p* < 0.001 indicate that significant differences between the PD and CT groups were detected with 2-tailed student *t*-test.

The concentrations of ATP, PCr, Pi, PE and GPC were also determined in PD and CT of Cohort II using the ^31^P MRS-MT data acquired without the γ-ATP resonance saturation, but similar SNR and spectral quality as Cohort I. As shown in Figure 3A,B, statistically significant decreases in the ATP, Pi and PE levels were detected again in the PD brains; and the pH values were also lower in PD (pH_PD_ = 7.031 ± 0.008, *n* = 11) than CT (pH_CT_ = 7.037 ± 0.008, *n* = 11) with *p* = 0.11. The individual values of ATP, Pi and PCr concentrations, intracellular pH, free [Mg^2+^] and PE/GPC ratios obtained from all participants in both cohorts are summarized in Figure 4. Statistically significant differences were detected in concentrations of ATP ([ATP]_PD_ = 2.62 ± 0.17 mM, [ATP]_CT_ = 2.82 ± 0.14 mM, *n* = 19, *p* = 0.0004), Pi ([Pi]_PD_ = 0.90 ± 0.12 mM, [Pi]_CT_ = 1.01 ± 0.11 mM, *n* = 19, *p* = 0.007), PE ([PE]_PD_ = 2.94 ± 0.49 mM, [PE]_CT_ = 3.32 ± 0.39 mM, *n* = 19, *p* = 0.012), and [PE]/[GPC] ratio ([PE/GPC]_PD_ = 1.29 ± 0.14, [PE/GPC]_CT_ = 1.45 ± 0.16, *n* = 19, *p* = 0.002), as well as in intracellular pH (pH_PD_ = 7.031 ± 0.006 mM, pH_CT_ = 7.036 ± 0.007 mM, *n* = 19, *p* = 0.021) between the PD and CT brains after combining the data from Cohorts I and II. Lower PCr levels were also observed in the PD brains ([PCr]_PD_ = 4.50 ± 0.53 mM, [PCr]_CT_ = 4.79 ± 0.34 mM, *n* = 19, *p* = 0.054), but due to large individual variations, the difference was slightly lower than the level of statistical significance.

### 2.3. Abnormal Bioenergetics in the Brain of PD Patients

To further evaluate the impaired energy metabolism in the occipital lobe of PD patients, we directly measured the forward reaction rate constant (k_f,ATPase_ and k_f,CK_) and cerebral ATP production rate (CMR_ATP_ and CMR_CK_) via the ATPase and CK reactions, respectively, using in vivo ^31^P MRS in combination with magnetization transfer (MT) technique (^31^P MRS-MT) [44] in Cohort II. As summarized in Figure 3C–E, we found that the forward rate constants of ATPase reaction were higher in PD patients (k_f,ATPase_^PD^ = 0.16 ± 0.03 s^−1^, *n* = 11) than that in age-sex matched controls (k_f,ATPase_^CT^ = 0.13 ± 0.02 s^−1^, *n* = 11) with a statistically significant *p* value of 0.013 (Figure 3C); and the mean metabolic rate of ATP production via the ATPase reaction was slightly higher in PD than CT although it did not reach a statistical significance (CMR_ATP_^PD^ = 7.50 ± 1.61 μmol/g/min, CMR_ATP_^CT^ = 6.65 ± 1.23 μmol/g/min, *n* = 11, and *p* = 0.18, Figure 3D). In contrast, the forward rate constant and ATP production rate via the CK reaction showed no difference between the two groups (k_f,CK_^PD^ = 0.35 ± 0.03 s^−1^, CMR_CK_^PD^ = 93.1 ± 7.0 μmol/g/min, *n* = 11; and k_f,CK_^CT^ = 0.35 ± 0.02 s^−1^, CMR_CK_^CT^ = 94.3 ± 6.4 μmol/g/min, *n* = 11; with *p* values of 1.0 and 0.43, respectively, calculated from Mann-Whitney U test, Figure 3C,E). The distributions of the k_f,ATPase_, k_f,CK_, CMR_ATP_ and CMR_CK_ values of the individual PD and CT in Cohort II are reported in Figure S2.

### 2.4. Pilot Test in Assessing Bioenergetic Effects of UDCA Treatment in PD Brains

To test the feasibility of monitoring treatment-induced bioenergetic changes, a subset of Cohort II-PD patients received a daily dose of oral UDCA for 6 weeks; and the same ^31^P MRS-MT measurements were performed before and after treatment. Three participants completed the pre- and post-UDCA scans with averaged blood concentrations of endogenous UDCA below 200 ng/mL prior to treatment and about 1600 ng/mL at end of the treatment. Their phosphorous metabolites concentration, intracellular pH, forward rate constant and cerebral metabolic rate of ATPase and CK reactions were determined before and after the 6-week UDCA treatment regimen as summarized in Figure S3.

In order to better understand the bioenergetic effects of the UDCA in the PD brain, we presented the measured parameters as the ratio of pre- and post-UDCA, and then compared them in parallel with the ratio of the PD and CT groups in Cohort II (see Figure S4 for details), assuming that the pre-UDCA condition and the PD group shared the same bioenergetic status. We found that the ATP, Pi and PCr concentrations, k_f,ATPase_, and CMR_ATP_ values in patient’s brain after the 6-week UDCA regimen were all shifted toward the levels of the CT brains. However, these changes were small relative to the PD vs. CT differences and did not reach statistical significance due to the limited number of subjects in this pilot clinical trial. In addition, small increases in the post-UDCA k_f,CK_ and CMR_CK_ values were also observed.

## 3. Discussion

Although it has long been suspected that mitochondrial dysfunction and energy failure causes neuronal death in a range of neurodegenerative diseases [4,5,6,45], it has been difficult to obtain direct and quantitative evidence that energy failure does occur in the brains of human patients. Previous in vivo ^31^P MRS studies of diseased brains have attempted to provide such proof by measuring steady-state levels of high-energy phosphates (i.e., ATP and PCr) and Pi, or their ^31^P signal (or concentration) ratios in different brain regions, but the results were inconsistent and elusive [18,21,23,25,26,27,28,29,30,31].

In this study, we utilized a 7T UHF human MRI scanner to quantitatively assess the bioenergetic status of visual cortex region in mild to moderate PD patients without dementia. Although the main pathology and clinical manifestations of PD are primarily associated with the substantia nigra (SN), PD is a systemic brain disorder and extra nigral areas of the brain, e.g., the cerebral cortex, could also develop signs of neurodegeneration owing to propagation of the disease [46], although the effects are usually less pronounced compared to the sub-cortical nuclei such as SN. It has been reported that PD patients with dementia developed abnormal metabolism in occipital lobe prior to onset of the dementia [47]. This supports the notion that abnormal metabolism and energetics due to mitochondrial dysfunction likely precede symptoms seen in patients with neurodegenerative diseases, particularly in brain region(s) without significant neuronal death [7]. If we could detect and quantify such subtle changes in the early stages of the disease using an advanced metabolic imaging technique, then it would become a powerful tool for accurately identifying disease onset, and monitoring its progression, or the efficacy of disease-modifying therapies.

With established MR imaging technology and the hardware currently available at 7T, we obtained ^31^P MRS data with excellent sensitivity and spectral quality (see Figure 1B for an example) from the human visual cortex, which ensured the reliability and accuracy of the measurements. In this study, the ^31^P MRS and ^31^P MRS-MT methods were used to independently evaluate two cohorts of PD and CT, and the trends of ATP, PCr, Pi, PE and pH changes were consistent in both cohorts. Overall, the decrease in intracellular ATP and Pi in the occipital lobe of PD patients was statistically significant (approximately 7% and 11%, respectively). However, there was no difference in the metabolite ratios of Pi/ATP, PCr/ATP and Pi/PCr between patients and controls, in part, because impaired metabolism in the patient brains resulted in parallel reductions of these phosphate metabolites (Figure 2A and Figure 3A). These findings highlighted the importance of absolute quantification of individual metabolites, which is more meaningful and sensitive to the assessment of brain phosphate contents and their changes than the ratio of different metabolites. The content of ATP and other phosphate compounds, and those of multiple metabolites may change under pathological conditions. Thus, the metabolite ratios only represent the relative levels of the two compounds. A difference in ratios cannot reveal the actual change in each metabolite; and the same ratio does not mean the content of each metabolite is constant.

In addition to quantifying the phosphate compounds involved in ATP metabolism, we also applied an in vivo NAD assay developed in our lab [37,38,39] to determine the oxidized and reduced NAD levels and NAD redox state in PD brains for the first time. We found that the intracellular NAD^+^ and total NAD contents in occipital lobes of the PD patients were significantly decreased, and their NAD^+^/NADH redox ratio was slightly lower compared to the control group. The validity of the in vivo NAD assay method has been evaluated and confirmed in both animal and human brains and at different magnetic fields [37,38,39,48]. Although NAD^+^ and NADH exist in different cellular compartment, all sub-cellular compartments contribute their signals detected by the MR-based NAD assay, with the majority signals from mitochondria and cytosol of the brain cells. Therefore, the NAD^+^/NADH values represent the intracellular NAD redox state of targeted brain tissues. Using the same method, it has been shown that cerebral NAD contents and redox ratios decline during normal aging [39,48]. Even so, NAD^+^ levels in the brains of PD patients were further reduced compared to age-matched controls. This observation is consistent with the pivotal role of NAD^+^ in cellular bioenergetics, genomic stability, mitochondrial homeostasis, adaptive stress responses, and cell survival (see the review article [49] and references cited therein). It has been widely accepted that NAD^+^ not only regulates the ATP energy metabolism through the NAD^+^/NADH redox reactions, but also serves as a sole substrate for different NAD^+^-dependent enzymes involved in various cellular signaling processes. The activity of these enzymes is sensitive to or regulated by cellular NAD^+^ levels, and NAD^+^ depletion has been reported in different neurological disorders [50,51,52,53,54]. For example, it has been shown that DNA damage activates the enzyme poly (ADP-ribose) polymerase 1 (PARP1); and higher PARP1 activity and lower NAD^+^ level are associated with ischemia, neuroinflammation, and neurodegenerative diseases. Moreover, intracellular NAD^+^ remains a crucial aspect in the pathogenesis/pathophysiology and treatment of Parkinson’s disease. In fact, a study of NAD^+^ and treatment in cellular models of the disease, e.g., patient-derived induced pluripotent stem cells, established that modulation of NAD metabolism might prove useful in the treatment of PD [55]. Therefore, the level of intracellular NAD may reflect brain tissue health. The non-invasively measured [NAD^+^], [NADH], [NAD_total_] and RX values provide valuable and sensitive biomarkers for assessing the pathophysiological condition of the human brain and potentially for monitoring therapeutic efficacy. This study demonstrates the first application of an in vivo NAD assay for investigating neurodegenerative diseases in human patients.

When profiling the cerebral phosphorous metabolites, we were able to determine the molar concentrations of PE and GPC, which represent the levels of precursors and intermediates of the phospholipid metabolism, respectively [56,57]. We found a statistically significant reduction in PE concentration and PE/GPC ratio but unchanged GPC levels in diseased brain, suggesting impaired membrane phospholipid anabolism and/or relatively increased membrane catabolism in the occipital lobe of PD patients. Meanwhile, in both Cohorts I & II, we observed lower intracellular pH in the PD brains as compared to age-matched controls, and this difference reached statistical significance when combining the data from the two cohorts. There has been age-related reductions in intracellular pH and membrane synthesis reported in healthy human brains [58]. Further reductions of pH and PE levels in age-matched diseased brains as shown herein suggest that the occipital lobe of the PD patients may have undergone more advanced aging processes than their healthy counterparts, even though this brain region has subtle or only late pathological changes in PD per the Braak Hypothesis [59].

Cortical thinning and subcortical atrophy that may occur at different stages in the pathogenesis of PD are well documented in the literature (e.g., [60,61]). The overall findings indicate that there is *no* significant volume change or atrophy in the occipital cortex of mild to moderate PD patients without cognitive impairment or dementia. Because the PD cohorts in our study showed similar cognitive functions as their healthy counterparts (see Table 1), it is unlikely that the reductions in the occipital ATP and other phosphorous metabolite levels as observed in this study could be explained by morphological change such as atrophy. It is noteworthy that our results were not corrected for the partial volume contribution of cerebrospinal fluid (CSF), which estimated to be ~10% or less [62,63]. However, even considering the partial volume of CSF, the amount of correction will be relatively small. It would, in fact, increase the concentrations of all metabolites in the PD and CT groups in parallel without changing the relationship between the two groups or the conclusions of this study.

To better understand the abnormal bioenergetics in the PD brains, we directly measured the forward rate constants and cerebral ATP production rates via ATPase and CK reactions using the in vivo ^31^P MRS-MT approach in Cohort II participants since both reactions contribute to changes in brain ATP levels. To our surprise, we found that the ATPase enzyme activity represented by the forward rate constant k_f,ATPase_ was higher in PD brains, while there was no significant difference in CMR_ATP_ values between the PD patients and healthy controls (see Figure 3 and Figure S2). On the other hand, the CK enzyme activity, expressed by the forward rate constant k_f,CK_, and CMR_CK_ values were the same in the two groups. These results suggested that there might be a cellular energy compensatory mechanism in this region of the PD brain, whereby brain cells attempt to maintain ATP homeostasis by increasing ATPase activity and ATP production. It has been shown in resting rat brains that ATP homeostasis was tightly regulated across a wide range of brain states, from light anesthesia to iso-electric state, by varying ATPase activity and CMR_ATP_. In contrast, CK activity and CMR_CK_ were relatively less sensitive to the changing brain states [64].

In a recently published functional ^31^P MRS-MT study, we further demonstrated distinctive and complementary roles of ATPase and CK reactions in supporting evoked neuronal activity and maintaining ATP homeostasis in healthy human brains. Our results showed that during physiological stimulation, the ATPase reaction dominated ATP energy production and supply, while the CK reaction played a complementary role in energy transportation and maintaining stable ATP levels [40]. Herein, we hypothesized that in the brains of PD patients, presumably DNA damage or other cellular defects modulate NAD^+^-dependent enzyme activities and reduce the intracellular NAD^+^ content, resulting in a decrease of steady-state ATP content in the brain. Therefore, upregulation of mitochondrial ATP synthase would likely occur to enhance ATP production and meet the energy requirements of the brain cells. However, we posit that as the disease progresses, the mismatch between the energy demand and ATP production will increase, eventually leading to energy failure and cell death [7]. Nevertheless, the pathogenesis and progression of neurodegenerative diseases vary across different brain regions at different disease stages [46,59]. For instance, similar changes may occur in the frontal or parietal lobe at much earlier time points in disease evolution, though this prediction requires a separate study to confirm. Further, different types of brain cells have different bioenergetic capacities and/or needs, so their ability to cope with activity-dependent fluctuations in bioenergetic demand could vary. Recent experimental evidences have provided clues as to why dopamine neurons in SN are particularly susceptible to the cellular dysfunctions commonly found in PD [65]. The data supports the idea that the heightened vulnerability of the nigral dopamine neurons can be directly attributed to their specific bioenergetic and morphological characteristics, i.e., these neurons have more complex axons and higher axonal mitochondria density. Consequently, they have a higher basal energy requirement and smaller energy reserve capacity, and therefore are increasingly vulnerable to the cellular stresses that impair mitochondrial energy production.

The ^31^P MRS-based metabolic imaging tools employed in this study and the results presented herein demonstrated the ability to non-invasively investigate the abnormal energy metabolism at different stages of human brain neurodegeneration. This advanced metabolic imaging technology, combined with a sophisticated quantification protocol that calibrates ATP concentration in each individual brain, can be applied to the same subject in different scan sessions. It can longitudinally assess disease progression and/or treatment response, especially to determine the degree of energy deficiency or the effectiveness of restoring brain energy metabolism. As a demonstration, we obtained pilot data in a small group of PD patients to examine the bioenergetic efficacy of UDCA treatment. UDCA is a naturally occurring hydrophilic bile acid and a FDA-approved drug for treating primary biliary cholangitis. Its anti-apoptotic and mitochondria-protective effects have been reported in various studies using PD or AD cell lines and preclinical animal models [41,42,43,66,67]. Particularly, UDCA displayed remarkable rescue effect for improving mitochondrial function [68,69,70,71] and increasing the cellular ATP level in a large-scale drug screen study [69]. The safety and tolerability of high doses of UDCA (up to 50 mg/kg/day) in patients with amyotrophic lateral sclerosis (ALS) have been demonstrated; and it has also been confirmed that UDCA can cross the blood brain barrier with measurable levels in the CSF that correlated with those from serum [72]. The pharmacokinetics, safety and tolerability of orally administered UDCA were evaluated in five PD patients, who are the subset of Cohort II in this study, and the results have recently been published [73].

The prior findings support the notion that UDCA has the potential to exert a central biological effect that improves brain mitochondrial function and cellular ATP availability in PD and may have beneficial disease modifying effects. Herein, we conducted the first in vivo measurement to assess the actual neuroenergetic response of human patients to UDCA treatment. The baseline and endpoint (i.e., 6-week) plasma UDCA concentrations data confirmed that UDCA is absorbed following oral administration and drug concentrations accumulated with the dosing regimen used in PD patients. The pilot ^31^P MRS-MT data suggested that orally administered UDCA may improve the intracellular ATP availability and normalize the ATPase activity and CMR_ATP_, and an enhanced role of creatine kinase reaction may partially contribute to the improved ATP availability after the UDCA treatment. Nevertheless, no conclusion can be draw from the limited pilot data; larger scale clinical studies are needed to confirm these observations and to further explore the potential of UDCA treatment for PD or other neurodegenerative diseases.

In recent decades, the prevalence of neurodegenerative diseases caused by extended lifespan has brought major challenges to our society. Accumulated evidence suggests that impaired brain energy metabolism, which subtly declined during aging and tightly associated with disease progression, may be a significant factor. Accordingly, therapeutic approaches based on brain energy rescue strategies have been explored (see review article [7] and references cited therein). To determine whether such approaches can truly improve the brain energetics and disease outcomes, metabolic imaging tools are essential. This study clearly demonstrated the capability of the proposed technology; however, in order to extend the current capability in research settings to broader clinical applications, further technique development is required. For example, although 7T clinical scanner has received FDA approval for brain applications, hardware and software support related to X-nuclei (non-proton) based metabolic imaging is still lacking. Also, commercial dual-frequency radiofrequency (RF) coils with optimal sensitivity and whole brain coverage are needed to target different brain regions affected by various brain disorders.

## 4. Materials and Methods

### 4.1. Participants and Study Design

The University of Minnesota Human Research Protection Program (UMN-HRPP) and Institutional Review Board (IRB) reviewed and approved the study protocol. Patients older than 18 years with medically stable, mild to moderate PD (supported by the Unified Parkinson Disease Rating Scale (UDPRS) and identified as stage I-II on the Hoehn and Yahr scale), and age- and sex-matched healthy controls (CT) were recruited for the study. Written informed consent was obtained from all participants prior to enrollment or before the MR imaging scans. All participants underwent a MR safety screen and the Montreal Cognitive Assessment (MoCA). Individuals who fail the safety screen, with unstable conditions, dementia or other neurological disorders, pregnant or lactating women, and those unable to adhere to study protocol for any other reason were excluded from participating in the study. All patients were off anti-Parkinson medication (~12 h) when they were scanned and UDPRS scores were obtained before resuming medication use.

Two cohorts of PD patients and their age-sex matched healthy controls were recruited in this study. In the first cohort of participants (Cohort I), we measured the profile of cerebral phosphorous metabolites using quantitative in vivo ^31^P MRS at 7T. Steady-state concentrations of energy phosphate compounds (ATP, PCr and Pi), phospholipid metabolites (PE and GPC), oxidized (NAD^+^) and reduced (NADH) NAD, thus, the total intracellular NAD content; as well as the NAD redox ratio (RX), intracellular pH and [Mg^2+^] were measured and compared between the PD (*n* = 8) and CT (*n* = 8) groups. In the second cohort of participants (Cohort II), we quantified the forward reaction rate constant (k_f_) and cerebral metabolic rate of ATP production via the ATPase and creatine kinase (CK) reactions in separate groups of PD (*n* = 11) and CT (*n* = 11) using in vivo ^31^P MRS-MT approach [44]. To evaluate ursodeoxycholic acid (UDCA), a naturally-occurring hydrophilic bile acid with known mitochondrial-protective effects, for its ability to improve brain bioenergetics in neurodegenerative patients, we recruited and enrolled five PD participants from Cohort II (Subset of Cohort II-PD) for a prospective 6-week open-label study of oral UDCA. Three patients (*n* = 3) were scanned before and after the 6-week UDCA trial using the same ^31^P MRS-MT protocol as Cohort II. The other two failed to complete the study due to scanner hardware problems.

### 4.2. UDCA Therapy and Dosing

UDCA tablets Urso (250 mg) and Urso Forte (500 mg) were purchased from Axcan Pharmaceuticals, Quebec City, QC, Canada [73]. The total daily dose divided into three approximately equal portions was given to each participant with increasing dosage at 15 mg/kg/day in week 1, 30 mg/kg/day in week 2 and 50 mg/kg/day in weeks 3–6. Each participant was instructed to take the study medication according to their individualized dosing schedule with meals three times per day. A weekly telephone survey was conducted to assess compliance and document any adverse events. In case of adverse events resulting from an escalated dose, participants were allowed to continue with the tolerated dose for the remainder of the study. Upon completion of the 6-week UDCA treatment, participants returned for the final study visit to receive their final dose of UDCA prior to the post-UDCA MR scan. Blood samples were collected from these patients prior to the MR scans to measure baseline or steady-state concentrations of UDCA and its conjugates using liquid chromatography-mass spectrometry (LC-MS) [74].

### 4.3. MR Data Acquisition

All ^1^H MRI and ^31^P MRS measurements were conducted on a whole-body/90-cm bore actively shielded 7T human scanner (Siemens MAGNETOM, Erlangen, Germany). Magnetic field (B_0_) shimming (up to 3rd order) was performed using Siemens 3D shimming sequence. A home-built RF probe consisting of passively decoupled ^1^H and ^31^P (5 cm diameter) dual surface coils was placed beneath the human occipital lobe for the acquisition of MR data. A small glass sphere containing a phosphorus reference (1.0 M solution of methylphosphonic acid) was fixed at the center of the ^31^P coil for RF power and pulse flip angle (FA) calibration. T_1_-weighted ^1^H anatomic images were acquired with TR/TE/TI = 3000/3.3/1500 ms (TR: repetition time, TE: echo time, TI: inversion time), nominal FA = 7°, and 1 mm isotropic resolution.

For Cohort I, ^31^P MR pulse-acquired spectra (number of transit (NT) = 320, TR = 3 s and FA = 84°) and 3D chemical shift imaging (CSI) data (field of view (FOV) = 12 × 12 × 9 cm^3^, phase-encoding matrix = 7 × 7 × 5, TR = 1.2 s, total NT = 896 and nominal FA = 68° using a (hard) RF pulse with 300 μs pulse width) were acquired. A 3D ^31^P-CSI data were also obtained after each human scan session from a head-sized spherical ATP phantom (containing 10 mM ATP, 10.3 mM [Mg^2+^] and ~50 mM [Na^+^] at pH of 7.0) with the same sample loading and position as the subject’s head to calibrate and quantify brain metabolite concentrations [40]. For Cohort II, ^31^P MRS-MT spectra with and without γ-ATP resonance saturation, respectively, were acquired by using the following parameters: 300 μs hard pulse with FA = 84° for excitation; RF magnetic field (B_1_) insensitive selective train to obliterate signal (BISTRO) [75] pulse (50 ms pulse width) train with 160 Hz saturation bandwidth and 1.37 s or 2.74 s saturation duration (T_sat_); and TR = 3 s and NT = 320. In each scan session, 3D ^31^P-CSI data of the brain and the ATP phantom with the same loading and position were acquired as described for Cohort I.

### 4.4. MR Data Analysis and Quantification

All ^31^P MR spectra were zero-filled; and a 10 Hz Lorentzian line broadening was applied before fast Fourier transformation to enhance the SNR. The AMARES (Advanced Method for Accurate, Robust, and Efficient Spectral fitting) time domain spectra fitting algorithm in the jMRUI software package (version 5.0) was used to analyze the ^31^P spectra [76,77]; and a custom-made MATLAB program based on the newly developed NAD assay method was used to analyze the spectra containing α-ATP, NAD^+^ and NADH peaks [38,39]. Compared with other quantitative methods, AMARES relies on non-linear least squares quantitative algorithms, combined with prior knowledge and constraints provided by users to improve quantitative accuracy [77]. The MR-based in vivo NAD assay uses a high-field MR scanner (7T in this study) to obtain the endogenous ^31^P MR signals of the NAD molecules in intact brains. It resolves the MR signal of NADH from that of NAD^+^ by taking advantage of their specific spectroscopic characteristics at a given magnetic field strength, thus, enables the quantification of submillimolar NAD^+^ and NADH contents in the human brain [38]. The integrals of the individual phosphorous metabolites from the spectral fitting were used to quantify their molar concentrations after correcting for saturation effects based on the T_1_, B_1_ and FA information. The T_1_ values of the human brain phosphorous metabolites at 7T were previously determined and employed in this study [33,34]. The intracellular pH values were determined from the chemical shift difference of Pi relative to PCr (δ_Pi−PCr_) based on the following equation:

pH = 6.75 + log_10_ [(δ_Pi-PCr_ − 3.27)/(5.63 − δ_Pi-PCr_)], (1)

and the free [Mg^2+^] content of the brain tissue was derived from the chemical shift differences of PCr to β-ATP using the subroutine in jMRUI software [76,78]. The resonance of PCr was set at −2.5 ppm and used as a chemical shift reference for other phosphorous compounds.

We have established a robust protocol for absolute quantification of phosphorous metabolite concentrations in human brains. The molar ATP concentration of each subject was determined first by comparing the ATP signals in the selected brain region (*I_brain_*) to that of ATP phantom (*I_phantom_*) using the 3D-CSI data acquired under fully relaxed condition in each scan session and following equation:

[ATP]_brain_ = [ATP]_phantom_ × *I_brain_* ÷ *I_phantom,_* (2)

Then, the [ATP]_brain_ was used as an internal reference for determining the molar concentration of other phosphorous metabolites in each brain with the correction of saturation effect [40]. The reported ATP concentration was calculated from an average of the γ- and α-ATP resonance signals.

A previously developed superfast magnetization saturation transfer method was employed to determine the reaction rate constant of the ATPase and CK reactions (k_f,ATPase_ and k_f,CK_) according to the equation:

M_c_/M_s_ ≈ 1 + k_f_ × T_1_^nom^, (3)

where M_c_ and M_s_ are controls; and γ-ATP saturated magnetizations of the Pi resonance for the ATPase reaction and the PCr resonance for the CK reaction acquired with a short TR under steady-state condition. T_1_^nom^ is a nominal T_1_ and its value can be derived via numerical simulation using modified Bloch–McConnell equations with known intrinsic T_1_ values of ATP, Pi and PCr and acquisition parameters at a given magnetic field strength [79,80]. The T_1_^nom^ values of the Pi and PCr were determined as 2.45 s and 3.14 s when T_sat_ = 2.74 s, or as 1.55 s and 1.91 s when T_sat_ = 1.37s, respectively. Thus, the corresponding cerebral metabolic rates of ATP production via ATPase (CMR_ATP_) and CK (CMR_CK_) reactions can be quantified as:

CMR_ATP_ = k_f,ATPase_ × [Pi], (4)

and

CMR_CK_ = k_f,CK_ × [PCr]. (5)

where [Pi] and [PCr] are the Pi and PCr concentrations (in mM unit) measured in the absence of γ-ATP saturation. The unit of CMR_ATP_ and CMR_CK_ becomes μmol/g/min after the conversion using the brain tissue density of 1.1 g/mL and s^−1^ for k_f_.

All results are presented as mean ± standard deviation (SD). Normality tests were performed on all reported data. Since most measured parameters have a normal distribution, parametric analysis (two-tailed Student *t*-test) was used for statistical comparisons between groups. For the few parameters that were slightly off the normal distribution, non-parametric analysis (i.e., two-tailed Mann-Whitney U test) was applied. A *p* value of <0.05 was considered statistically significant.

## 5. Conclusions

By performing metabolic and energetic assessments of PD patients and age-sex matched healthy controls, we have provided compelling evidences showing abnormal energy metabolism in cortical brain region of the PD patients. A comprehensive matrix of bioenergetic and neurophysiological parameters expressed in *absolute* units or scales was obtained non-invasively for the first time from human patients and healthy volunteers. This included the concentrations of major phosphorous metabolites related to ATP energy metabolism and phospholipid metabolism, oxidized and reduced NAD contents, and intracellular pH and NAD redox ratio, as well as the ATPase and CK enzyme activities and the corresponding ATP production rates. Through these truly quantitative measurements, different brains or brain regions can be directly compared at different times or under different conditions. These results provided important new knowledge of metabolic and energetic alterations associated with neurodegeneration and accelerated aging in human patients. The advanced ^31^P MRS-based metabolic imaging technology as described herein bridges the gap between cellular metabolism studies of biological samples and intact human brains. Therefore, we believe it can be used to better understand neurodegenerative diseases and other brain disorders, monitor disease progression and possibly evaluate patients’ responses to investigational treatments.

## Figures and Tables

**Table 1:** Subject Characteristics.

	Cohort I		Cohort II		Subset of Cohort II-PD	
	PD	CT	PD	CT	Pre-UDCA	Post-UDCA
Subject Number	8	8	11	11	3	3
Gender	4M/4F	4M/4F	5M/6F	5M/6F	2M/1F	2M/1F
Age (years)	62 ± 7	60 ± 8	64 ± 8	61 ± 8	67 ± 11	
UDPRS Score	24 ± 15	-	36 ± 11	-	40 ± 10	37 ± 12
MoCA Score	27 ± 3	29 ± 2	28 ± 2	29 ± 1	27 ± 2	28 ± 2

PD: Parkinson’s disease patient; CT: healthy control; M: male; F: females; UDPRS: Unified Parkinson Disease Rating Scale; and MoCA: Montreal Cognitive Assessment. All data are presented as Mean ± SD.

## Data Availability

The data presented in this study are available on reasonable request from the corresponding author. The data are not publicly available due to ongoing clinical trial.

## Supplementary Materials

The following are available online at https://www.mdpi.com/2218-1989/11/3/145/s1, Figure S1: Distribution of the intracellular NAD^+^, NADH and total NAD contents, as well as the NAD^+^/NADH redox ratio determined in individual PD patients and healthy controls of Cohort I, Figure S2: Distribution of the forward reaction rate constant of k_f,ATPase_ and k_f,CK_, and cerebral metabolic rate of ATP production CMR_ATP_ and CMR_CK_ in individual PD patients and healthy controls of Cohort II, Figure S3: Summary of phosphorous metabolites concentration, intracellular pH, forward rate constant and cerebral metabolic rate of ATPase and CK reactions measured in a subset of the Cohort II-PD brains before and after 6-weeks of oral UDCA treatment, Figure S4: Bioenergetic changes between the PD and control (CT) groups of Cohort II, and the pre- and post-UDCA conditions in subset of Cohort II-PD, represented by the PD/CT or pre-/post-UDCA ratios of metabolites concentration, forward rate constant and metabolic rate of ATPase and CK reactions, respectively.
