# Supplementary material for: Quantitative Assessment of Occipital Metabolic and Energetic Changes in Parkinson’s Patients, Using In Vivo 31P MRS-Based Metabolic Imaging at 7T
Source: Metabolites. 2021 Mar 1;11(3):145. doi: 10.3390/metabo11030145 (PMC8000945; doi:10.3390/metabo11030145)
Supplement: Supplementary file 1 [file metabolites-11-00145-s001.pdf]

Quantitative Assessment of Occipital Metabolic and Energetic Changes in Parkinson's Patients using *In Vivo* ^{31}P MRS-based Metabolic Imaging at 7T

[1]Xiao-Hong Zhu*, [1]Byeong-Yeul Lee, [2]Paul Tuite, [3]Lisa Coles, [3]Abhishek G. Sathe, [4]Chi Chen, [3]Jim Cloyd, [5]Walter C. Low, [6]Clifford J. Steer and [1]Wei Chen*

[1]Center for Magnetic Resonance Research, Department of Radiology; [2]Department of Neurology; [3]Department of Experimental and Clinical Pharmacology; [4]Department of Food Science and Nutrition; [5]Department of Neurosurgery; [6]Departments of Medicine and Genetics, Cell Biology and Development; University of Minnesota, Minneapolis, MN, USA

*Correspondence: Xiao-Hong Zhu, PhD

or Wei Chen, PhD

Supplemental Material (four figures)**:**

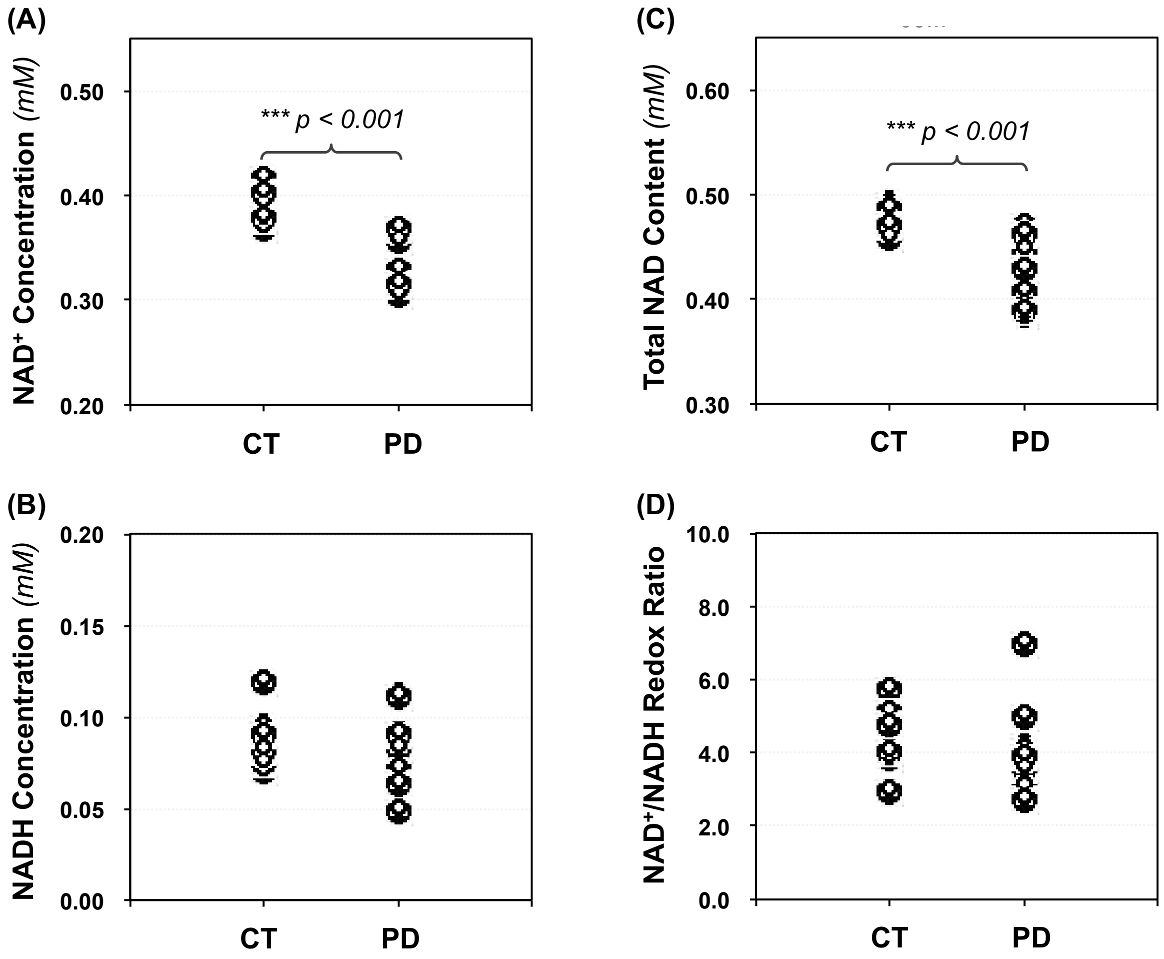

Figure S1: Distribution of the intracellular NAD^+ (**A**), NADH (**B**) and total NAD (**C**) contents, as well as the NAD^+/NADH redox ratio (**D**) determined in individual PD patients (n=8) and healthy controls (n=8) of ***Cohort I***. *** $p<0.001$ indicates that significant differences between the PD and CT groups were detected with 2-tailed Student t-test.

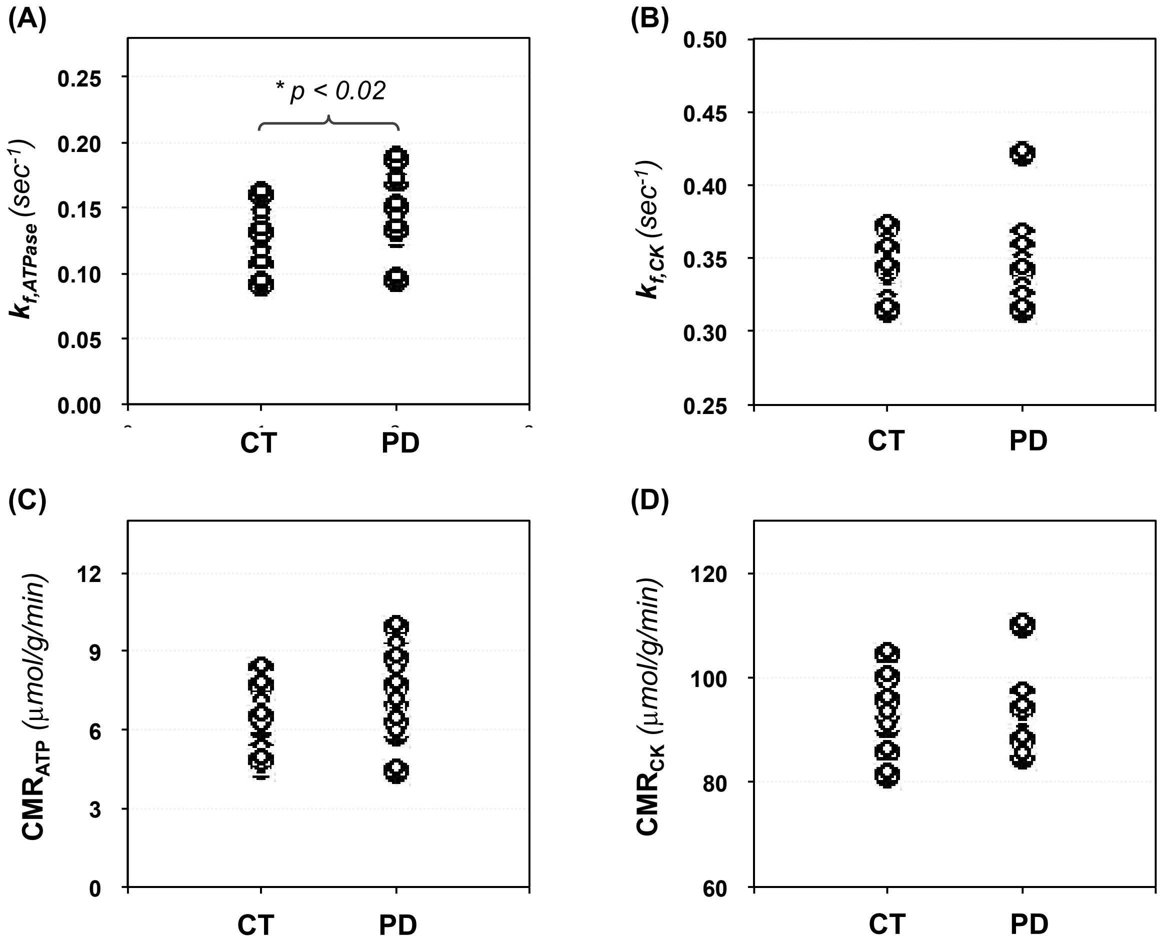

Figure S2: Distribution of the forward reaction rate constant of $k_{f,ATPase}$ (**A**) and $k_{f,CK}$ (**B**) and cerebral metabolic rate of ATP production CMR_{ATP} (**C**) and CMR_{CK} (**D**) in individual PD patients (n=11) and healthy controls (n=11) of ***Cohort II***. * p<0.02 indicates that significant differences between the PD and CT groups were detected with 2-tailed Student t-test.

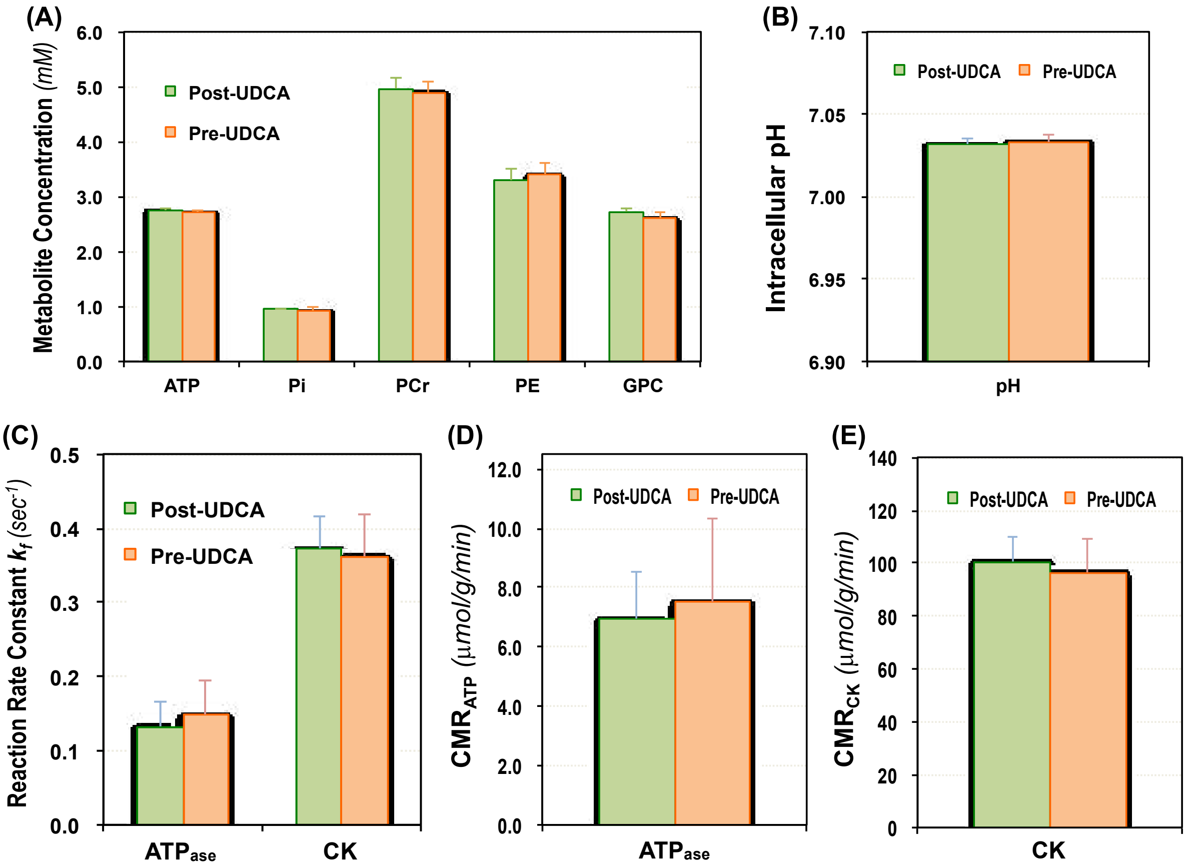

Figure S3: Summary of phosphorous metabolites concentration (**A**), intracellular pH (**B**), forward rate constant (**C**) and cerebral metabolic rate of ATPase (**D**) and CK (**E**) reactions measured in a subset of the ***Cohort II-PD*** brains (n=3) before and after 6-week of oral UDCA treatment. All data are presented as Mean ± SD.

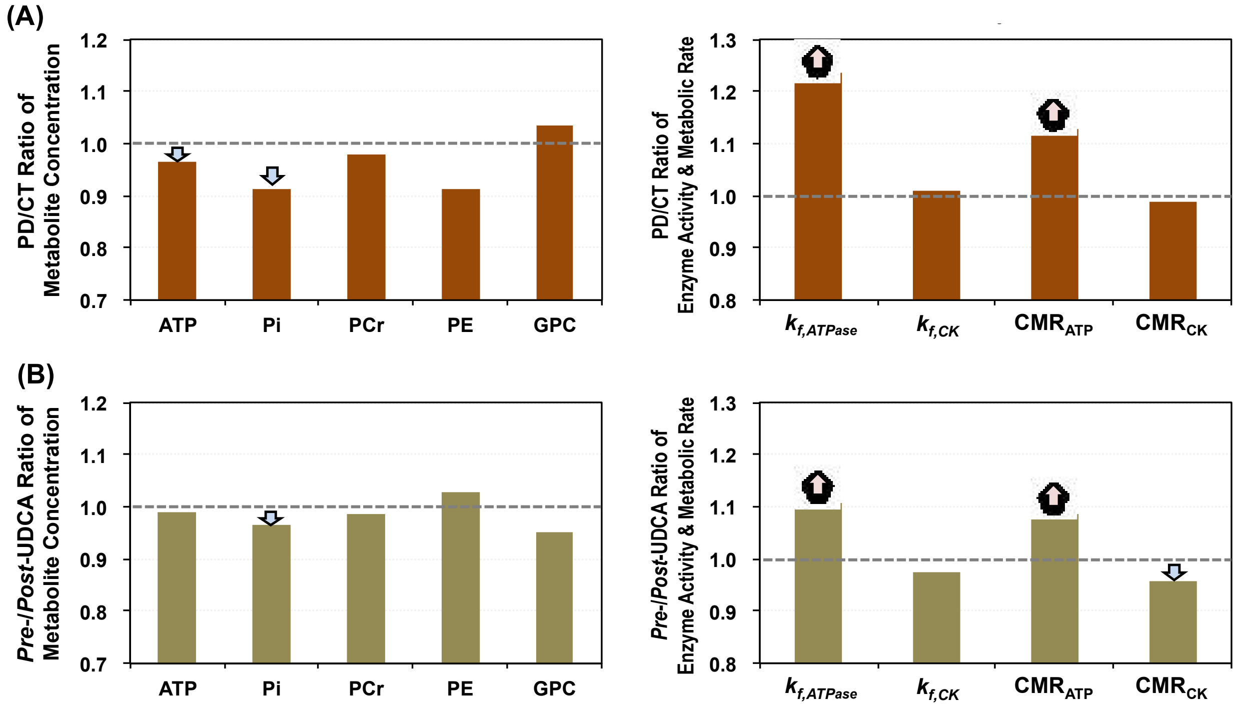

Figure S4: (**A**) Bioenergetic changes between the PD (n=11) and control (n=11) groups of ***Cohort II***, and (**B**) the pre- and post-UDCA conditions in *subset of* ***Cohort II-PD*** (n=3), represented by the *PD/CT* or *pre-/post-UDCA ratios* of metabolites concentration, forward rate constant and metabolic rate of ATPase and CK reactions, respectively. The red and blue arrows indicate the elevated and decreased values of the particular metabolic parameter of interest, while the dashed lines mark unchanged parameter values between the two groups or conditions.
